# Impact of Fetal Growth Restriction on the Neonatal Microglial Proteome in the Rat

**DOI:** 10.3390/nu13113719

**Published:** 2021-10-22

**Authors:** Manuela Zinni, Julien Pansiot, Marina Colella, Valérie Faivre, Andrée Delahaye-Duriez, François Guillonneau, Johanna Bruce, Virginie Salnot, Jérôme Mairesse, Marit Knoop, Marie-Laure Possovre, Daniel Vaiman, Olivier Baud

**Affiliations:** 1 Faculté de Médecine, Inserm UMR 1141 NeuroDiderot, Université de Paris, F-75019 Paris, France; manuela.zinni@inserm.fr (M.Z.); julien.pansiot@inserm.fr (J.P.); colella.marina@gmail.com (M.C.); valerie.faivre@inserm.fr (V.F.); andree.delahaye@inserm.fr (A.D.-D.); 2 UFR de Santé, Médecine et Biologie Humaine, Université Sorbonne Paris Nord, F-93000 Bobigny, France; 3 Institut Cochin, INSERM, CNRS, 3P5 Proteom’IC Facility, Université de Paris, 22 rue Méchain, F-75014 Paris, France; francois.guillonneau@parisdescartes.fr (F.G.); johanna.bruce@parisdescartes.fr (J.B.); virginie.salnot@parisdescartes.fr (V.S.); 4 Laboratory of Child Growth and Development, University of Geneva, 1205 Geneva, Switzerland; jerome.mairesse@inserm.fr (J.M.); marit.knoop@unige.ch (M.K.); marie-laure.possovre@unige.ch (M.-L.P.); 5 Institut Cochin, Inserm U1016, UMR8104 CNRS, F-75014 Paris, France; daniel.vaiman@inserm.fr; 6 Division of Neonatology and Pediatric Intensive Care, Children’s University Hospital of Geneva, 1205 Geneva, Switzerland

**Keywords:** microglia, IUGR, inflammation, proteome, brain development, oxidative stress

## Abstract

Microglial activation is a key modulator of brain vulnerability in response to intra-uterine growth restriction (IUGR). However, the consequences of IUGR on microglial development and the microglial proteome are still unknown. We used a model of IUGR induced by a gestational low-protein diet (LPD) in rats. Microglia, isolated from control and growth-restricted animals at P1 and P4, showed significant changes in the proteome between the two groups. The expression of protein sets associated with fetal growth, inflammation, and the immune response were significantly enriched in LPD microglia at P1 and P4. Interestingly, upregulation of protein sets associated with the oxidative stress response and reactive oxygen species production was observed at P4 but not P1. During development, inflammation-associated proteins were upregulated between P1 and P4 in both control and LPD microglia. By contrast, proteins associated with DNA repair and senescence pathways were upregulated in only LPD microglia. Similarly, protein sets involved in protein retrograde transport were significantly downregulated in only LPD microglia. Overall, these data demonstrate significant and multiple effects of LPD-induced IUGR on the developmental program of microglial cells, leading to an abnormal proteome within the first postnatal days.

## 1. Introduction

Intra-uterine growth restriction (IUGR) and prematurity are the two leading complications of human pregnancy: every year, 30 million infants are delivered after IUGR and 15 million are born preterm [1,2]. These conditions are recognized to be the two main contributors to neonatal brain injury responsible for the neurodevelopmental disorders that affect more than nine million children each year [3,4,5,6,7,8]. Perinatal systemic inflammation is known to be associated with both IUGR and prematurity, and early exposure to inflammation has been identified as an important risk factor for the development of neurological impairment later in life [9,10].

The brain inflammatory response is mainly mediated by microglial cells, the resident macrophages of the central nervous system (CNS). Microglia play a key role in multiple functions of the brain. Microglia can acquire distinct phenotypes in response to perinatal inflammatory events, not only contributing to the restoration of brain homeostasis but also supporting repair and regulating important physiological processes, including synaptic pruning [11,12,13,14,15,16]. Response to injury in the developing brain is usually associated with but not restricted to an inflammatory response of microglia [17].

Due to their important role in the modulation of brain development and the regulation of brain homeostasis, the control of microglial activity during the early phase of life appears to be a critical factor for the protection of the developing brain and prevention of the emergence of neurodevelopmental disorders. Indeed, recent evidence suggests that aberrant microglial activity is a critical factor for the development of cerebral palsy and autism spectrum disorders [18,19,20,21].

Exposure to a low-protein diet (LPD) during gestation in rodents is a recognized preclinical model to induce IUGR and its developmental consequences on the immature brain [22,23]. Animals exposed to an antenatal LPD are characterized by brain alterations, including defective myelination and abnormal brain connectivity. These anatomical and functional abnormalities are associated with the polarization of microglia reactivity toward a pro-inflammatory phenotype, observable at both the transcriptional and morphological level [24,25,26,27].

The early phase of life is a critical window for brain plasticity and adaptation to a challenging environment. Studies already published on the microglial transcriptome have shown developmental heterogeneity of microglia associated with a dynamically changing transcriptomic phenotype during development [28,29,30]. Microglial proteome studies previously focused on comparing juveniles and adults [31] or characterized the microglial proteome in animal models of adult neurodegenerative disease [32,33,34]. However, potential changes in the microglial proteome during the early postnatal period in animals subjected to antenatal injury are not yet known. 

Indeed, previous studies on the effects of IUGR have focused only on the total brain proteome or specific brain structures, including the hippocampus and hypothalamus [35,36,37,38]. Although a previous study showed an effect of IUGR on the induction of inflammation-associated proteins in rats exposed to prenatal food restriction [38], none have investigated the direct consequences of IUGR on the microglial proteome early in development.

We aimed, therefore, to characterize the consequence of IUGR on microglial development and investigate the consequences of exposure to IUGR on the microglial proteome during the first postnatal days using a bioinformatics approach associated with functional assays. 

## 2. Materials and Methods

### 2.1. Animals and Diets

All experiments were carried out in compliance with Inserm ethical rules and approved by the institutional review board (Ministry of Higher Education and Scientific Research, Directorate-General for Research and Innovation, Paris, France), in accordance with the European Communities Council Directive 2010/63/EU. Briefly, Sprague–Dawley dams (Janvier Labs, Le Genest-Saint-Isle, France) were randomly divided into two groups according to their diet: a 22% (normal) protein diet (control, CTRL) or an isocaloric 9% protein diet (LPD) from the day of conception until delivery, as previously described [39]. The weekly food intake, protein intake, and body growth of the pregnant rats fed with normal diet or LPD are described in the Supplementary Figure S1. Pups of both sexes from multiple litters were used in the study to avoid a litter effect.

### 2.2. Magnetic Sorting of Microglial Cells

Primary microglial cells were sorted at P1 and P4 using MACS MicroBead Technology column-based magnetic cell isolation with an anti-CD11b/c antibody (Miltenyi Biotec, Bergisch Gladbach, Germany), as previously described [27]. A slight modification of the protocol was applied in the experiments dedicated to proteomic analysis. Four brains per sample at P1 and three brains per sample at P4 were pooled in both the CTRL and LPD experimental groups. The elution was performed using cold 1X PBS and an additional wash step with cold 1× PBS was performed after the final centrifugation to remove contaminating bovine serum albumin (BSA). 

### 2.3. Primary Microglial Cell Culture and Immunocytochemistry

Microglial cells at P4 were isolated and cultured as previously reported [27]. Briefly, microglia were cultured in DMEM/F-12 media (Thermo Fisher Scientific, Waltham, MA, USA) supplemented with 10% FBS and 1% penicillin/streptomycin. After fixation with 4% PFA, cells were stained with goat anti-Iba1 (1:500; ab5076, Abcam, Cambridge, UK), mouse anti-GFAP (1:500, G3893, Sigma Aldrich, St. Louis, MO, USA), rabbit anti-NeuN (1:500, ABN78, Millipore, Burlington, MA, USA), rabbit anti-Olig2 (1:200, 18953, Immuno-Biological Laboratories, Minneapolis, MN, USA), and DAPI (1:10,000). The following day, cells were incubated with secondary antibodies coupled to the green (Alexa Fluor 488) and red fluorescence markers (Alexa Fluor 555) (1:500) for 1 h at room temperature. Cells were analyzed using a fluorescent microscope (Nikon Eclipse Ti-E, Nikon Instruments, Melville, NY, USA), and the number of cells stained for Iba1, GFAP, NeuN, and Olig2 were counted. Six wells and five fields per well were analyzed (50–80 cells per well), and the cell number was normalized to the DAPI staining. Results are expressed as the percentage relative to the number of cells.

### 2.4. Measurements of Reactive Oxygen Species (ROS) Production by Luminometry

Microglial cells were magnetically sorted from P4 control and LPD rats and immediately processed for reactive oxygen species (ROS) measurement. In detail, 20,000 microglial cells were resuspended in Hanks’ balanced salt solution (HBSS; Invitrogen, Thermo Fisher Life Technologies, Waltham, MA, USA) to a final volume of 160 µL and incubated with luminol (50 µM, Sigma, St. Louis, MO, USA) for 10 min at 37 °C in the dark. As a functional test, we compared a control to a sample that was stimulated with 10^−7^ M phorbol 12-myristate 13-acetate (PMA, Sigma, St. Louis, MO, USA). PMA is a protein kinase C agonist linked to ROS production, in particular in white blood cells, expressing NADPH oxidase, such as microglia [40]. Each condition was assayed in duplicate. Cells were then immediately analyzed using a luminometer (Centro LB 960; Berthold Technologies, Bad Wildbad, Germany) in a 96-well plate. Each measurement cycle consisted of recording the signal for 1 s in each well. The duration of one cycle, i.e., the interval between two measurements in each well, was 1 or 2 min, depending on the total number of wells. The signal was recorded over a 20 min period and reported as relative light units (RLUs). Results are expressed as the area under the curve (AUC) of luminescence over the 20 min.

### 2.5. Sample Preparation for Proteomic Analysis

Cells were solubilized in lysis buffer (2% SDS, 50 mM Tris-HCl, pH 8.0, 10 mM TCEP, 50 mM chloroacetamide) and heated for 5 min at 95 °C. Protein extracts were clarified by centrifugation at 21,000× *g* for 1 h at 4 °C. The protein concentration of the supernatant was estimated and normalized using image intensity integration of a Coomassie blue G250-colored SDS-PAGE gel loaded with the same volume of each lysate. Tryptic peptides for bottom-up experiments were obtained using S-Trap micro spin columns according to the manufacturer’s protocol (ProtiFi, Huntington, NY, USA). Briefly, 30 μg of protein from the above lysates was diluted to 100 μL in lysis buffer. Proteins were digested for 14 h at 37 °C with 1 µg sequencing-grade trypsin (Promega, Charbonnières-les-Bains, France). The S-Trap micro spin column was used according to the manufacturer’s protocol. After speed-vacuum drying, eluted peptides were solubilized in 2% trifluoroacetic acid (TFA) and fractionated by strong cationic exchange (SCX) Stage-Tips, essentially as described [41].

### 2.6. Liquid Chromatography–Coupled Mass Spectrometry Analysis (LC-MS)

LC-MS analyses were performed on a Dionex U3000 RSLC nano-LC system (Thermo Fisher Scientific, Waltham, MA, USA) coupled to a TIMS-TOF Pro mass spectrometer (Bruker Daltonik GmbH, Bremen, Germany). After drying, five fractions of the peptides from the SCX Stage-Tips were solubilized in 10 μL 0.1% TFA containing 10% acetonitrile (ACN). One microliter was loaded, concentrated, and washed for 3 min on a C18 reverse-phase precolumn (3 μm particle size, 100 Å pore size, 75 μm inner diameter, 2 cm in length, from Thermo Fisher Scientific, Waltham, MA, USA). Peptides were separated on an Aurora C18 reverse-phase resin (1.6 μm particle size, 100 Å pore size, 75 μm inner diameter, 25 cm in length), connected to a Captive nanoSpray Ionization module (IonOpticks, Middle Camberwell, Australia) with a 120 min run time and a gradient ranging from 99% solvent A, containing 0.1% formic acid in milliQ-grade H_2_O, to 40% solvent B, containing 80% acetonitrile and 0.085% formic acid in mQH_2_O. The mass spectrometer acquired data throughout the elution process and operated in the DDA PASEF mode, with a 1.1 s/cycle, with the timed ion mobility spectrometry (TIMS) mode enabled and a data-dependent scheme with full MS scans in PASEF mode. This enabled recurrent loop analysis of a maximum of the 120 most intense nLC-eluting peptides, which were CID fragmented between each full scan every 1.1 s. The ion accumulation and ramp times in the dual TIMS analyzer were set to 50 ms each, and the ion mobility range was set from 1/K0 = 0.6 vs. cm^−2^ to 1.6 vs. cm^−2^. Precursor ions for MS/MS analysis were isolated in positive mode with the PASEF mode set to « on » in the 100–1700 *m*/*z* range by synchronizing quadrupole switching events with the precursor elution profile from the TIMS device. The cycle duty time was set to 100%, accommodating as many MSMS in the PASEF frame as possible. Singly charged precursor ions were excluded from the TIMS stage by tuning the TIMS using Otof control software (Bruker Daltonik GmbH, Bremen, Germany). Precursors for MS/MS were picked from an intensity threshold of 2500 arbitrary units (a.u.) and re-sequenced until reaching a ‘target value’ of 20,000 a.u., taking into account the dynamic exclusion of a 0.40 s elution gap.

### 2.7. Protein Quantification and Comparison

Mass spectrometry data were analyzed using MaxQuant version 1.6.17 (Max-Planck Institute of Biochemistry, Computational Systems Biochemistry, Heidelberg, Germany) [42]. The database used was a concatenation of *Rattus norvegicus* sequences from the UniProt and-SwissProt databases (release 2020-10) and a list of contaminant sequences from MaxQuant. The enzyme specificity was that for trypsin. The mass tolerances for the precursors and fragments were set to 20 ppm. Carbamidomethylation of cysteines was set as a permanent modification, and acetylation of the N-terminus and oxidation of methionines were set as variable modifications. A second peptide search was allowed, and the minimal length of peptides was set to seven amino acids. The false discovery rate (FDR) was maintained at <1% for both peptides and proteins. Label-free protein quantification (LFQ) was performed using both unique and razor peptides. At least two such peptides were required for LFQ. The “match between runs” (MBR) option was allowed with a match time window of 0.7 min and an alignment time window of 20 min. For differential analysis, LFQ results from MaxQuant were quality-checked using PTXQC [43] and then imported into Perseus software version 1.6.14 (Max-Planck Institute of Biochemistry) [44]. Reverse and contaminant proteins were excluded from the bioinformatic analysis.

### 2.8. Bioinformatic Analysis

Protein set enrichment analysis was performed using GSEA version 4.1.0 (Broad institute, Cambridge, MA, USA). Proteins were ranked accordingly to the signal-to-noise metrics and enrichment was based on the weighted Kolmogorov–Smirnov-like statistic. A phenotype permutation was performed 1000 times for the analysis, and GSEA was used to identify protein sets against the Reactome pathway database. Reactome pathways defined by an FDR < 0.25 and a *p* value < 0.05 were considered significant. Proteins differentially expressed were analyzed using the WEB-based GEne SeT AnaLysis Toolkit (http://www.webgestalt.org, accessed on 12 october 2021). An over-representation analysis (ORA) against the Reactome, the Gene Ontology (GO), and the Mammalian Phenotype Ontology database was performed to identify the enriched Reactome pathways, biological processes, and possibly related phenotypes. To note, this last database is defined in Webgestalt for mice only and not for rat, but assuming a strong similarity between the physiological processes between the two species, we used in this case ‘*Mus musculus’* as an organism of interest. Our specific microglial proteome dataset was used as a reference protein set for the analysis.

### 2.9. Statistical Analysis

All data are reported as means ± standard error of the mean (SEM). Statistical analysis of all data was performed using GraphPad PRISM version 9.0 (San Diego, CA, USA). Body-weight data were analyzed using Student’s unpaired *t*-test. Two-way ANOVA, followed by a Newman–Keuls multiple comparisons test, was applied to the analysis of ROS assay data. Significance was set at *p* < 0.05 for all tests. Sample reproducibility was analyzed by principal component analysis (PCA) performed on Log2 transformed LFQ values applying a filter of 100% valid values. Based on the PCA results, two outlier samples (1 CTRL P1 and 1 CTRL P4) were excluded from the analysis. Heat maps were built using Morpheus (https://software.broadinstitute.org/morpheus, accessed on 12 october 2021), and clustering was analyzed using raw values and columns using Pearson correlations on *Z*-score-transformed values. Venn diagrams were generated using the GeneVenn tool (http://genevenn.sourceforge.net, accessed on 12 october 2021). Statistical details, including sample size information, are reported in Supplementary Table S1.

## 3. Results

### 3.1. Evaluation of Purity after Cell Sorting and Sample Clustering

**Figure 1:**
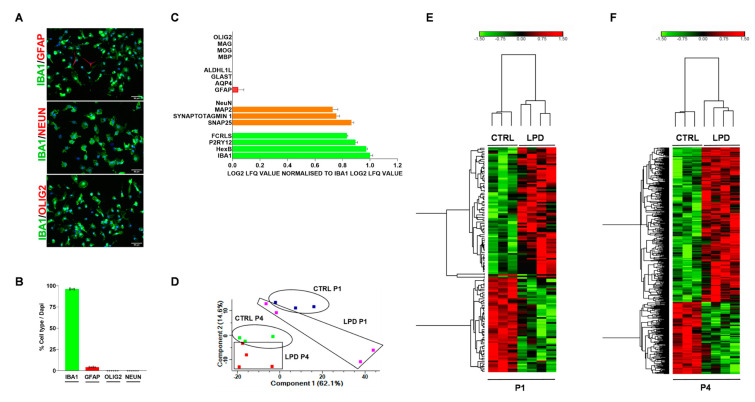
Assessment of cell purity after sorting and sample clustering. Microglial cells were stained with Iba1 (green), either GFAP, Olig2, or NeuN (red), and DAPI (blue). (**A**) Representative photomicrographs at ×20 magnification (scale bar = 50 μm). (**B**) Percentage of IBA1+, GFAP+, Olig2+, and NeuN+ cells normalized to DAPI staining. (**C**) Expression of microglial (FcRLS, P2RY12, HexB, Iba1), neuronal (NeuN, MAP2, Synaptotagmin1, SNAP25), astrocyte (ALDHAL1L, GLAST, AQP4, GFAP), and oligodendrocyte (Olig2, MAG, MOG, MBP) cell markers in the proteome dataset. (**D**) Principal component analysis. (**E**,**F**) Heat maps showing the clustering in (**E**) CTRL and LPD at P1 and (**F**) CTRL and LPD at P4. Upregulated proteins are depicted in red and downregulated proteins in green.

We assessed the purity of sorted microglial cell samples using cell-type-specific markers. The highest proportion of labeled cells in culture were Iba1+ (96.15% ± 0.42%) (Figure 1A,B). Immunostaining showed the absence of oligodendrocytes and neurons, but the GFAP marker identified a small proportion of astrocytes (4.13% ± 0.29%) (Figure 1A,B). We investigated the proteomic profile of our samples by evaluating the expression of the various cell-type-markers in CTRL and LPD microglia at P1 and P4. The microglia markers IBA1, HexB, P2RY12, and FCRLS were highly expressed (Figure 1C). We also observed low expression of GFAP, consistent with the low astrocyte contamination observed in the primary microglia culture (Figure 1C). Oligodendrocyte-specific markers were not detectable. On the contrary, gene expression of certain neuronal markers, including SNAP25, SYNAPTOTAGMIN 1, and MAP2, was observed but without immunocytochemical evidence of neuronal contamination (Figure 1C). No difference in cell marker expression has been evidenced among experimental groups, meaning that the purity of sorted microglial cell samples appears similar.

We next evaluated the ability of principal component analysis (PCA) to discriminate between the four experimental groups of the microglial proteome. PCA showed good separation between the CTRL and LPD groups at both P1 and P4 (Figure 1D). In addition, there was clear discrimination between P1 and P4 samples in both the CTRL and LPD microglial cell samples (Figure 1D). The clustering reported in the heat map confirms the homogeneity of the various samples in both experimental groups (Figure 1E,F).

### 3.2. The Microglial Proteome Early after Birth Reflects the Growth Restriction following LPD Exposure

**Figure 2:**
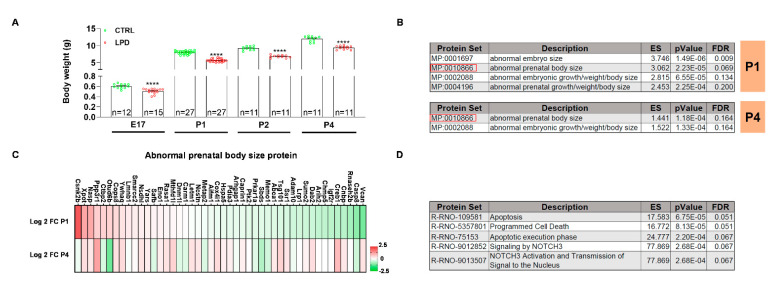
The microglial proteome reflects the low body weight observed in LPD animals early during their development. (**A**) Body weight (g) of CTRL and LPD animals at the embryonic stage (E17) and the postnatal days P1, P2, and P4. Data (Mean ± SEM), **** *p* < 0.0001 vs. CTRL. (**B**) ORA analysis of differentially expressed proteins between CTRL and LPD at P1 and P4 performed against the mammalian phenotype database. (**C**) Log2 fold change (FC) of proteins associated with the abnormal prenatal body size protein set. Reported Log2 FCs were calculated for the following comparisons LPD P1 vs. CTRL P1 and LPD P4 vs. CTRL P4. Upregulated proteins are depicted in red and downregulated proteins in green. (**D**) ORA analysis of proteins associated with the abnormal prenatal body size protein set against the Reactome pathway database.

Growth restriction is one of the major features observed in animals exposed to antenatal LPD. Indeed, exposure to LPD from E1 to E17 significantly reduced the body weight of the embryos (t_25_ = 5.437, *p* < 0.0001) (Figure 2A). There was also a significant reduction in body weight for LPD animals during the first postnatal day (P1 = t_52_ = 18.35, *p* < 0.0001; P2 = t_20_ = 11.64, *p* < 0.0001; P4 = t_20_ = 9.02, *p* < 0.0001).

We performed a protein set enrichment analysis for differentially expressed (DE) proteins between CTRL and LPD microglia at P1 and P4 against the Mammalian Phenotype Ontology database. Protein sets associated with the body weight and body size were significantly enriched in LPD microglia at P1 (Figure 2B). We observed similar, but less pronounced, protein set enrichment in P4 LPD microglia (Figure 2B). We then focused on the protein set “abnormal prenatal body size (MP: 0010866)” enriched in both P1 and P4 microglia. Log2 fold change (FC) values showed the deregulation of MP: 0010866 proteins at both P1 and P4, either down or upregulated in microglia by LPD exposure (Figure 2C). The major deregulation of this protein set by LPD exposure observed at P1 was reduced at P4 (Figure 2C). The MP: 0010866-associated proteins were analyzed against the Reactome pathway database to better understand their biological role. The analysis showed these proteins to be involved in the regulation of apoptotic processes and the NOTCH3 signaling pathway (Figure 2D).

### 3.3. Protein Sets Associated with Inflammation Are Deregulated in the LPD Microglial Proteome

**Figure 3:**
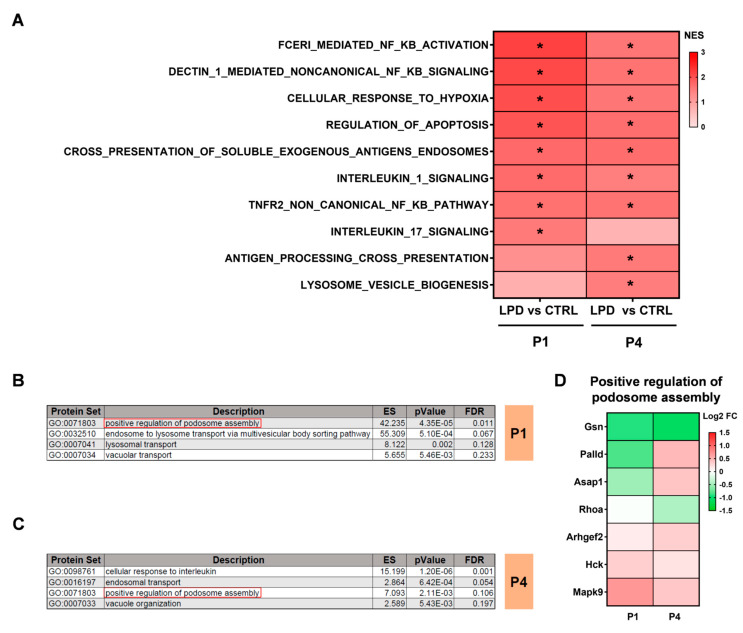
Protein sets associated with inflammation are deregulated in the LPD microglial proteome. (**A**) GSEA analysis performed against the Reactome pathway database. Upregulated protein sets in LPD P1 vs. CTRL P1 and LPD P4 vs. CTRL P4 are reported, * *p* < 0.05 vs. CTRL. (**B**,**C**) ORA analysis of differentially expressed proteins between (**B**) CTRL and LPD at P1 and (**C**) CTRL and LPD at P4 performed against the GO database. (**D**) Log2 FC of proteins associated with the positive regulation of podosome assembly. Upregulated proteins are depicted in red and downregulated proteins in green.

A deregulated inflammatory response at the transcriptomic level has been previously reported in microglial cells isolated from LPD animals during the first postnatal days [25,26]. We performed a GSEA analysis against the Reactome database focused on Reactome pathways involved in inflammation and significantly upregulated in LPD microglia. Exposure to an LPD induced significant upregulation of proteins associated with the “NFkB signaling” and “interleukin 1 signaling” (R-RNO-9020702) pathways in both P1 and P4 LPD microglia. Similarly, we observed increased expression of proteins involved in the “cellular response to hypoxia” (R-RNO-1234174) and the “regulation of apoptosis” (R-RNO-169911) in the P1 and P4 LPD microglial proteome (Figure 3A).

There was significant upregulation of the Reactome pathway “interleukin 17 signaling” (R-RNO-448424) in LPD microglia relative to CTRL only at P1, whereas the expression of proteins associated with “antigen processing cross presentation” (R-RNO-1236975) and “lysosome vesicle biogenesis” (R-RNO-432720) increased only at P4 in LPD microglia (Figure 3A).

Finally, proteins differentially expressed between CTRL and LPD microglia at P1 and P4 were analyzed against the gene ontology (GO) database targeting biological processes associated with inflammation. Among the significantly enriched biological processes, 8% were found to be associated with the development of an immune response and antigen processing. Proteins involved in the regulation of “lysosomal transport” (GO: 0007041) and “transport between endosome and lysosome” (GO: 0032510) were overexpressed in the LPD P1 microglial proteome relative to that of CTRL microglia (Figure 3B). Similarly, there was enrichment of “endosomal transport” (GO: 0016192) and the “cellular response to interleukins” (GO: 0098761) protein sets in LPD microglia at P4 (Figure 3B,C). “Positive regulation of podosome assembly” (GO: 0071803) was found to be a common enriched biological process in both P1 and P4 microglia sorted from LPD pups (Figure 3D). Podosomes are important structures for the migration of microglia and extracellular matrix degradation by microglial cells.

### 3.4. Protein Sets Associated with Oxidative Stress and Cellular Response to Oxidative Stress Are Enriched in LPD Microglia

**Figure 4:**
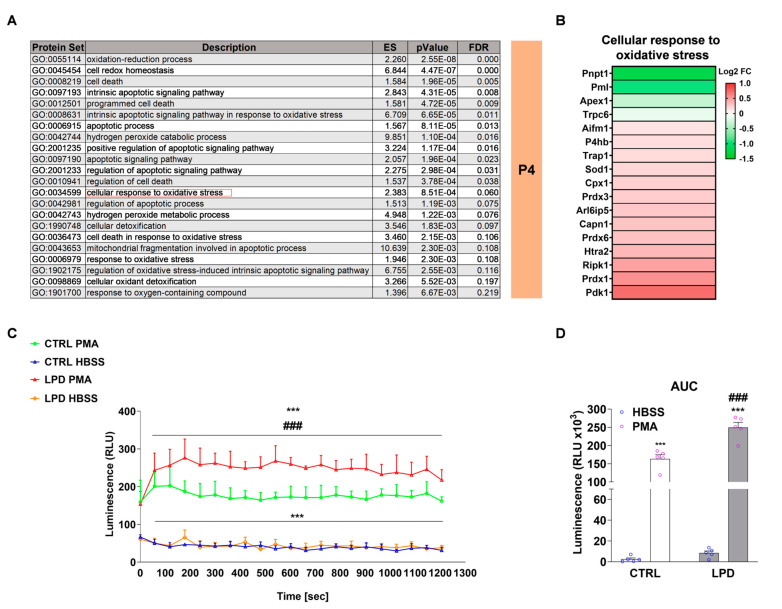
Protein sets associated with oxidative stress and cellular response to oxidative stress are enriched in the LPD microglial proteome. (**A**) ORA analysis of DE expressed proteins between CTRL and LPD at P4 performed against the GO database. (**B**) Log2 FC of proteins associated with the cellular response to oxidative stress. Upregulated proteins are depicted in red and downregulated proteins in green. (**C**,**D**) ROS assay: (**C**) Relative light unit (RLU) values and the (**D**) area under the curve (AUC) corresponding to the total RLU signal measured over 20 min are reported. Data (Mean ± SEM), *** *p* < 0.001 vs. respective HBSS, ### *p* < 0.001 vs. CTRL.

The production of ROS is a key event in the inflammatory response of microglia. We observed significant enrichment of protein sets associated with oxidative stress and the cellular response to oxidative stress, including sets involved in the regulation of cell death and apoptotic processes, in LPD microglia relative to CTRL in P4 animals (Figure 4A). Proteins associated with the “cellular response to oxidative stress” (GO: 0034599) biological process were significantly deregulated in LPD microglia, with 13 of 17 proteins upregulated (Figure 4B).

We next performed a functional assay to further confirm the increased expression of proteins involved in the oxidative stress response observed in the LPD microglial proteome. CTRL and LPD microglia were stimulated, or not stimulated, with PMA immediately after magnetic sorting to quantify the basal (HBSS) and PMA-induced ROS production. LPD exposure did not modify basal ROS levels. PMA exposure significantly increased ROS release in both CTRL and LPD microglia (interaction: F_(1, 16)_ = 19.35, *p* = 0.0004; CTRL:HBSS vs. CTRL:PMA: *p* < 0.001; LPD:HBSS vs. LPD:PMA: *p* < 0.001) but the effect was significantly potentiated by pre-exposure to an antenatal LPD (CTRL:PMA vs. LPD:PMA: *p* < 0.001) (Figure 4C,D).

### 3.5. The Early Developmental Changes of the Microglial Proteome Are Modified by Antenatal LPD Exposure

**Figure 5:**
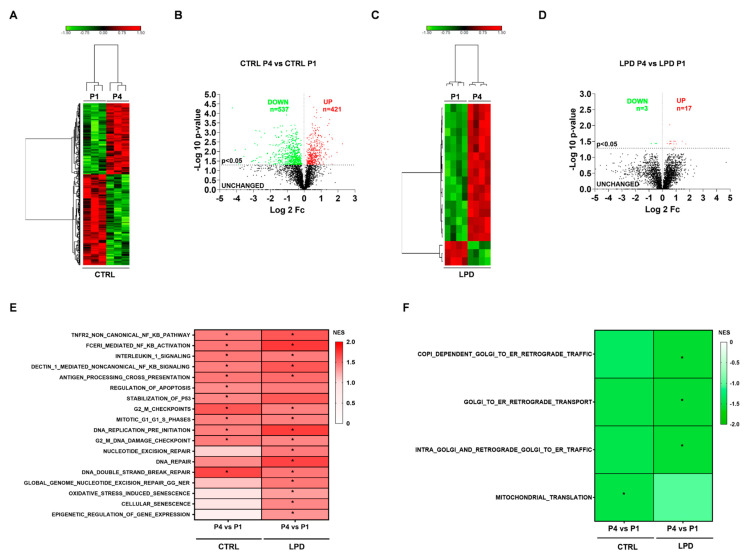
Early developmental changes in the microglial proteome are modified by exposure to LPD. (**A**,**C**) Heat maps showing the clustering in (**A**) CTRL at P1 and P4 and (**C**) LPD at P1 and P4. (**B**,**D**) Volcano plot showing differentially expressed proteins between (**B**) CTRL P4 and CTRL P1 and (**D**) LPD P4 and LPD P1. Upregulated proteins are depicted in red, downregulated proteins in green, and unchanged in black. (**E**) GSEA analysis performed against the Reactome pathway database. Upregulated protein sets in CTRL P4 vs. CTRL P1 and LPD P4 vs. LPD P1 are reported. (**F**) GSEA analysis performed against the Reactome pathway database. Downregulated protein sets in CTRL P4 vs. CTRL P1 and LPD P4 vs. LPD P1 are reported. * *p* < 0.05 vs. respective P1 time point.

We assessed the developmental changes in the microglial proteome during the first postnatal days and the influence of antenatal LPD exposure. First, physiological changes induced by age were shown by comparing CTRL P1 and P4. A heat map showed good discrimination and sample reproducibility between the two time points (Figure 5A). Age strongly modified the microglial proteome in CTRL animals, with 421 proteins upregulated and 537 downregulated at P4 relative to P1 (Figure 5B). Similar to the CTRL microglia, the heat map showed clustering in LPD microglia sorted at P1 and P4 (Figure 5C). The analysis of proteins differentially expressed showed only 17 proteins to be significantly upregulated and three downregulated at P4 relative to P1 in LPD microglia (Figure 5D).

We found pathways associated with inflammation to be significantly upregulated at P4 relative to P1 by GSEA analysis against the Reactome pathway database. Proteins involved in “NFkB signaling”, “interleukin 1 signaling” (R-RNO-9020702), and “antigen processing cross presentation” (R-RNO-1236975) were upregulated in both CTRL and LPD microglia at P4. Similarly, proteins associated with cell-cycle-related pathways were found to be upregulated in P4 microglia relative to P1 for both CTRL and LPD microglia (Figure 5E). Aside from these similarities, upregulation of several other Reactome pathways, including “nucleotide excision repair” (R-RNO-1236975), “DNA repair” (R-RNO-73894), “global genome nucleotide excision repair gg ner” (R-RNO-5696399), “oxidative stress induced senescence” (R-RNO-2559580), “cellular senescence” (R-RNO-2559583), and “epigenetic regulation of gene expression“(R-RNO-212165), occurred only in LPD P4 microglia (Figure 5E).

Among Reactome pathways downregulated during development, we found that “mitochondrial translation” was significantly downregulated in P4 CTRL microglia relative to P1 CTRL microglia (Figure 5F). LPD exposure significantly downregulated the expression of proteins included in pathways related to retrograde transport from the Golgi apparatus to the endoplasmic reticulum (ER) (Figure 5F).

**Figure 6:**
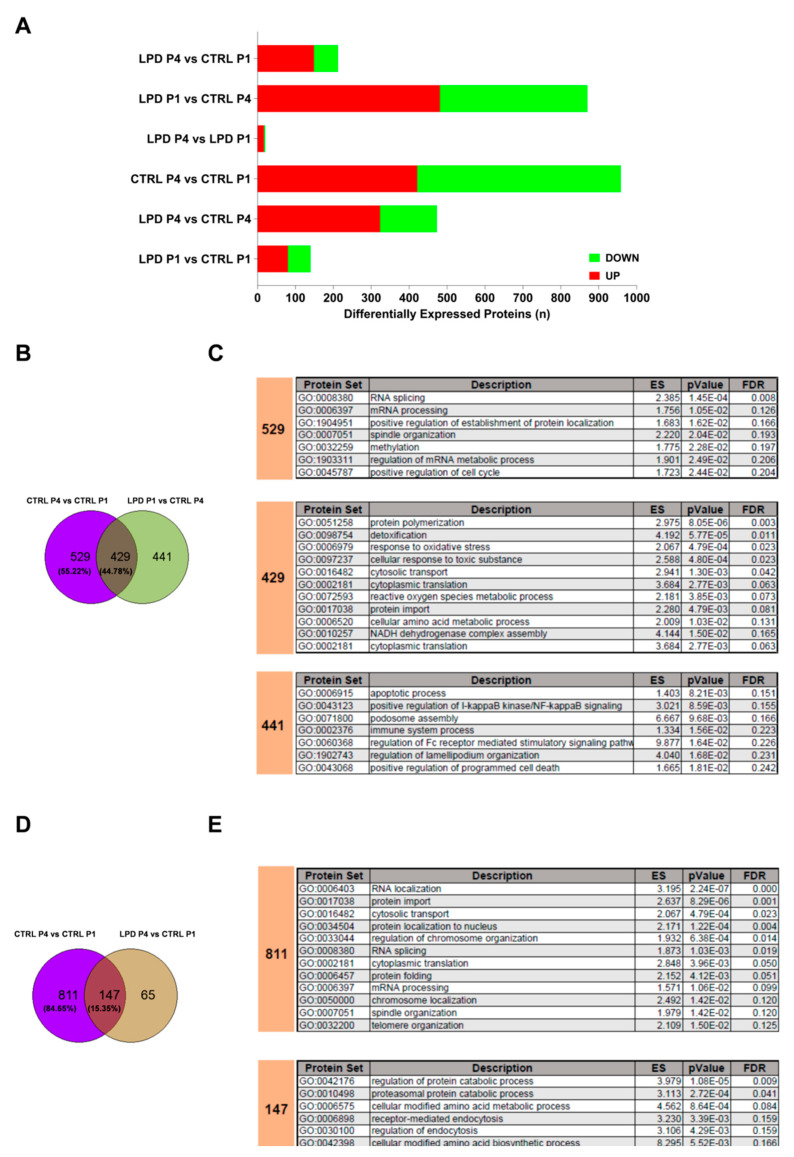
LPD exposure may induce a blockade of microglial maturation. (**A**) Graph showing the number of proteins differentially expressed between the experimental groups. Upregulated proteins are depicted in red and downregulated proteins in green. (**B**) GeneVenn diagram showing the percentage of common differentially expressed proteins between CTRL P4 and CTRL P1 and between LPD P1 and CTRL P4 and (**C**) results of the analysis against the GO database. (**D**) GeneVenn diagram showing the percentage of common differentially expressed proteins between CTRL P4 and CTRL P1 and between LPD P4 and CTRL P1 and (**E**) results of the analysis against the GO database.

The between-group difference in protein expression may not only explain LPD-induced brain alterations during the first postnatal days but also provide novel insights about the impact of LPD on microglial maturation. Indeed, we observed a much stronger effect of age in CTRL microglia (significant differential regulation of 928 proteins) relative to LPD microglia (significant differential regulation of only 20 proteins) (Figure 5B,D and Figure 6A). While only 140 proteins were differentially expressed in LPD microglia relative to CTRL microglial at P1, LPD exposure reduced the developmental changes observed between P1 and P4 in CTRL animals (only 243 differentially expressed proteins between LPD P4 and CTRL P1 versus 928 between CTRL P4 and CTRL P1). Hence, antenatal exposure to LPD appears to disrupt the normal maturation of developing microglia between P1 and P4 (Figure 6A).

We next compared protein sets that were significantly modified when the proteomes of CTRL P4 animals were compared to those of either CTRL P1 or LPD P1 animals. This analysis was performed to assess the early effect of antenatal LPD exposure at P1 on subsequent microglial maturation. Among the 928 proteins differentially expressed between P1 and P4 in CTRL animals, 429 (44.78%) were found in common with those differentially expressed in CTRL P4 relative to LPD P1 (Figure 6B). By contrast, 529 proteins were found to no longer be modulated by age in animals subjected to antenatal LPD, and 441 proteins were newly modulated. GO analysis showed that proteins regulated during the developmental maturation of microglia only in CTRL but not in LPD-exposed animals have a role in the regulation of key biological processes for normal cell physiology, including those involved in mRNA splicing/processing, post-transduction protein processing, and regulation of the cell cycle (Figure 6C). Biological processes significantly enriched between P1 and P4 in both CTRL and LPD animals have a role in the response of the cell to oxidative stress. Finally, proteins only differentially expressed between LPD P1 and CTRL P4 have a role in the immune system response, apoptosis, and cell morphology (Figure 6C).

We performed a similar analysis to assess the interaction of LPD exposure on microglial maturation between P1 and P4. The expression of a large number of proteins was selectively modified during normal development in CTRL microglia (*n* = 811, 84.65% of the total modified). The expression of only 147 proteins (15.35% of total physiologically modified) was commonly modified in both CTRL and LPD P4 microglia relative to the CTRL microglia at P1 (Figure 6D). Biological process analysis confirmed the significant enrichment of key processes associated with normal cell physiology and identified processes linked to cell metabolism and cell trafficking for proteins for which developmental regulation was not affected by LPD (Figure 6E). No significant enrichment of any biological process reached an FDR < 0.25 when the 65 proteins exclusively modified in LPD microglia at P4 relative to CTRL microglia at P1 were studied by GO analysis.

## 4. Discussion

Perinatal inflammation associated with prematurity and IUGR is an important risk factor for the emergence of neurodevelopmental disorders [9,10]. A reduced nutrient supply to the fetus due to maternal undernutrition during gestation is the most common cause of IUGR [45,46]. We developed an animal model of IUGR by exposing pregnant rats to an isocaloric and hypoprotein diet (LPD) during gestation. LPD animals are characterized by structural and functional alterations defined by brain hypomyelination and alterations of brain connectivity [25]. Such brain alterations are associated with an abnormal brain inflammatory response linked to increased microglial reactivity shown both in vivo and in vitro [24,25,26].

The results reported here provide novel insights into understanding the effects of LPD exposure on the microglial phenotype. We focused on the developmental program of microglial cells, and our data show alterations of the microglial proteome within the first postnatal days, with abnormal expression of protein sets associated with the immune system and inflammation-related immune response.

The evaluation of cell purity is a critical point to assess before the proteomic analysis of samples. In accordance with results previously reported at the RNA level [27], microglia represented the main cell type and there was only minor contamination with astrocytes and oligodendrocytes in the proteome dataset generated from CTRL and LPD microglia at P1 and P4. In contrast, some neuronal proteins were found to be expressed in our proteome dataset, although immunohistochemical analysis did not show neuronal contamination. This apparent discrepancy between the in vitro results and the proteome dataset can be explained by synaptic pruning and the phagocytosis of dying and living neurons by microglia [11,12,13,14,15,16,47].

IUGR is clinically defined by low body weight [48]. In accordance with our previous observations, animals exposed to an LPD diet showed a lower body weight at embryonic stage E17 and during the first postnatal week, despite a relative increase in the carbohydrate intake in the LPD group, in particular during the first two antenatal weeks. A recent study pointed out that mRNA expressions of markers of microglia infiltration were increased in the brain of the mice fed with high-carbohydrate diets [49]. In our model, analysis of the microglial proteome showed a microglial signature that reflects the IUGR phenotype observed in LPD animals. Protein sets associated with body weight were, indeed, significantly enriched in LPD microglia at both time points, and we observed deregulation of proteins involved in the modulation of body weight in both P1 and P4 LPD microglia, with a tendency toward reversal of the effect observed at P1 in P4 microglia. The analysis revealed a role of the differentially expressed proteins in the modulation of important biological processes, such as apoptosis and modulation of the NOTCH3 signaling transduction pathway, which has been recently reported to be essential for the positive modulation of the pro-inflammatory transcription factor NFkB in macrophages [50]. This is the first evidence of an association between the IUGR phenotype and the microglial proteome. Further studies are necessary to understand the details of this association and to address the question of a potential sex dependence of these changes in microglial proteome.

IUGR is a main inductor factor of the neonatal brain injury responsible for the neurodevelopmental disorders observed in infants, and data evidenced a significant increase of circulating cytokines at 7 days and 14 days after birth in infants born after IUGR [51,52]. Although a direct correlation has not been established yet, the postnatal systemic pro-inflammatory condition observed in IUGR babies could be responsible, at least in part, for the neurodevelopmental impairment detected in childhood in IUGR individuals. Oxidative stress and an abnormal ROS production have been consistently reported in IUGR in both clinical and animal studies [53,54,55]. In addition, an abnormal inflammatory response in the brain has been extensively described in preclinical studies conducted in a model of IUGR [24,25,26,27,56,57], and the exacerbated microglia reactivity was evidenced to be associated with brain hypomyelination and with the development of behavioral alterations [25,26]. Several studies have focused on the brain proteome alterations induced by IUGR [35,36,37,38] and the recent study conducted by Potiris et al. highlighted a role of IUGR in the induction of proteins associated with inflammation [38]. We show, for the first time, a specific microglial proteomic signature induced by IUGR early in development. LPD exposure has been previously associated to a shift toward a pro-inflammatory phenotype [24,25], and the results here presented confirm the relation between IUGR and microglia phenotype. The inflammatory response mediated by microglia is a multifactorial process involving not only the secretion of cytokines and chemokines but also the release of ROS [58]. Exacerbated and deregulated ROS production by microglia is an important hallmark for the development and progression of neurodegenerative disease [59]. We show the enrichment of protein sets related to the oxidative stress response, associated with an increase in the production of ROS in response to stimulation. The effect was limited to P4 LPD microglia, suggesting increased sensitivity of proteins associated with oxidative stress to the effect of LPD at this developmental stage.

Studies have shown that the microglial transcriptome dynamically changes during development, defining multiple microglial signatures [28,29,30], identifying three developmental stages: (i) early microglia (until E14), (ii) pre-microglia (from E14 to the early postnatal weeks), and (iii) adult microglia [60]. In addition, at least nine transcriptionally distinct microglial states were reported to be associated with developmental changes throughout the lifespan and to show heterogeneity, in particular, early in development [30]. Environmental factors can modify microglial development and induce a shift or an acceleration of their maturation. A shift to an advanced maturational stage has, indeed, been recently reported at the transcriptomic level in a poly(I:C) animal model of maternal immune activation [60] and in young mice injected with LPS [61]. Exposure to LPD modified the physiological developmental profile of microglia. Only a few proteins were, indeed, differentially expressed in P4 LPD microglia relative to P1, and we observed upregulation of protein sets associated with inflammation, in addition to downregulation of proteins involved in Golgi–ER transport. Interestingly, proteins involved in the execution of the DNA repair associated process and events associated with cellular senescence were upregulated only in LPD, suggesting a unique LPD signature linked to the necessity to counteract the exposure to greater stress. There are two possible explanations for such an alteration in microglial maturation: (i) an acceleration of microglial maturation or (ii) blockade of microglial maturation at the P4 stage.

## 5. Conclusions

Overall, the data presented here demonstrate a significant effect of LPD-induced IUGR on the developmental program of microglial cells, leading to an abnormal proteome within the first postnatal days. Further studies using single-cell RNA-seq are needed to distinguish between the blockage of microglial maturation or specific changes of microglial cell clusters induced by exposure to LPD during gestation.

## Data Availability

The mass spectrometry proteomics data have been deposited to the ProteomeXchange Consortium (http://proteomecentral.proteomexchange.org, accessed on 12 October 2021) via the PRIDE partner repository with the dataset identifier PXD028950.

## Supplementary Materials

The following are available online at https://www.mdpi.com/article/10.3390/nu13113719/s1, Table S1: Statistical details; Figure S1: Weekly food intake (**A**), protein (**B**) and body weight (**C**) in pregnant rats fed with normal (*n* = 17) and low protein (*n* = 20) diets.
