# Supplementary material for: Impact of Fetal Growth Restriction on the Neonatal Microglial Proteome in the Rat
Source: Nutrients. 2021 Oct 22;13(11):3719. doi: 10.3390/nu13113719 (PMC8624771; doi:10.3390/nu13113719)

Figure 1	Variable	Number of well	Number of cells/well
1B	% of cell stained / dapi	6	50-80

		Student's t-test		N=number of animals	
Figure 2	Variable	t-values	p-values	CTRL	LPD
2A	body weight	E17 t_{25}=5.437	p<0.0001	12	15
		P1 t_{52}=18.35	p<0.0001	27	27
		P2 t_{20}=11.64	p<0.0001	11	11
		P4 t_{20}=9.02	p<0.0001	11	11

		LPD vs CTRL P1		LPD vs CTRL P4	
	Reactome pathway	Nominal pValue	FDR	Nominal pValue	FDR
Figure 3A	FCERI_MEDIATED_NF_KB_ACTIVATION	0,000	0,000	0,000	0,052
	DECTIN_1_MEDIATED_NONCANONICAL_NF_KB_SIGNALING	0,000	0,000	0,000	0,145
	CELLULAR_RESPONSE_TO_HYPOXIA	0,000	0,000	0,000	0,047
	REGULATION_OF_APOPTOSIS	0,000	0,000	0,000	0,1269
	CROSS_PRESENTATION_OF_SOLUBLE_EXOGENOUS_ANTIGENS_ENDOSOMES	0,000	0,015	0,000	0,092
	INTERLEUKIN_1_SIGNALING	0,000	0,014	0,000	0,163
	TNFR2_NON_CANONICAL_NF_KB_PATHWAY	0,000	0,038	0,000	0,1373
	INTERLEUKIN_17_SIGNALING	0,000	0,065	0,667	0,976
	ANTIGEN_PROCESSING_CROSS_PRESENTATION	0,750	1,000	0,000	0,0643
	LYSOSOME_VESICLE_BIOGENESIS	1,000	1,000	0,000	0,106

		2 Way Anova		N=number of animals	
Figure 4	Variable	F-values	p-values	CTRL	LPD
4C	ROS	Interaction:$F_{(60, 320)}$= 3.808	P<0.0001	5 HBSS, 5 PMA	5 HBSS, 5 PMA
4D	ROS AUC	Interaction: $F_{(1, 16)}$=19.35	P=0.0004	5 HBSS, 5 PMA	5 HBSS, 5 PMA

		CTRL P4 vs CTRL P1		LPD P4 vs LPD P1	
	Reactome pathway	Nominal pValue	FDR	Nominal pValue	FDR
Figure 5E	TNFR2_NON_CANONICAL_NF_KB_PATHWAY	0,000	0,047	0,000	0,177
	FCERI_MEDIATED_NF_KB_ACTIVATION	0,000	0,000	0,000	0,215
	INTERLEUKIN_1_SIGNALING	0,000	0,026	0,000	0,210
	DECTIN_1_MEDIATED_NONCANONICAL_NF_KB_SIGNALING	0,000	0,050	0,000	0,184
	ANTIGEN_PROCESSING_CROSS_PRESENTATION	0,000	0,220	0,000	0,152
	REGULATION_OF_APOPTOSIS	0,000	0,055	0,182	0,900
	STABILIZATION_OF_P53	0,000	0,058	0,200	0,900
	G2_M_CHECKPOINTS	0,000	0,041	0,000	0,148
	MITOTIC_G1_G1_S_PHASES	0,000	0,127	0,000	0,159
	DNA_REPLICATION_PRE_INITIATION	0,000	0,097	0,020	0,148
	G2_M_DNA_DAMAGE_CHECKPOINT	0,000	0,098	0,031	0,161
	NUCLEOTIDE_EXCISION_REPAIR	0,154	0,436	0,000	0,167
	DNA_REPAIR	0,111	0,390	0,000	0,000
	DNA_DOUBLE_STRAND_BREAK_REPAIR	0,000	0,031	0,000	0,191
	GLOBAL_GENOME_NUCLEOTIDE_EXCISION_REPAIR_GG_NER	0,133	0,373	0,000	0,157
	OXIDATIVE_STRESS_INDUCED_SENESCENCE	0,785	0,885	0,000	0,109
	CELLULAR_SENESCENCE	0,923	0,943	0,000	0,168
	EPIGENETIC_REGULATION_OF_GENE_EXPRESSION	1,000	1,000	0,037	0,229

		CTRL P4 vs CTRL P1		LPD P4 vs LPD P1	
	Reactome pathway	Nominal pValue	FDR	Nominal pValue	FDR
Figure 5F	COPI_DEPENDENT_GOLGI_TO_ER_RETROGRADE_TRAFFIC	0,583	0,807	0,000	0,078
	GOLGI_TO_ER_RETROGRADE_TRANSPORT	0,583	0,841	0,000	0,078
	INTRA_GOLGI_AND_RETROGRADE_GOLGI_TO_ER_TRAFFIC	0,417	0,813	0,000	0,230
	MITOCHONDRIAL_TRANSLATION	0,000	0,238	0,615	0,884

<u>Supplementary Figure 1</u>: Weekly food intake (A), protein intake (B) and body weight (C) in pregnant rats fed with normal (N=17) and low protein (N=20) diets.

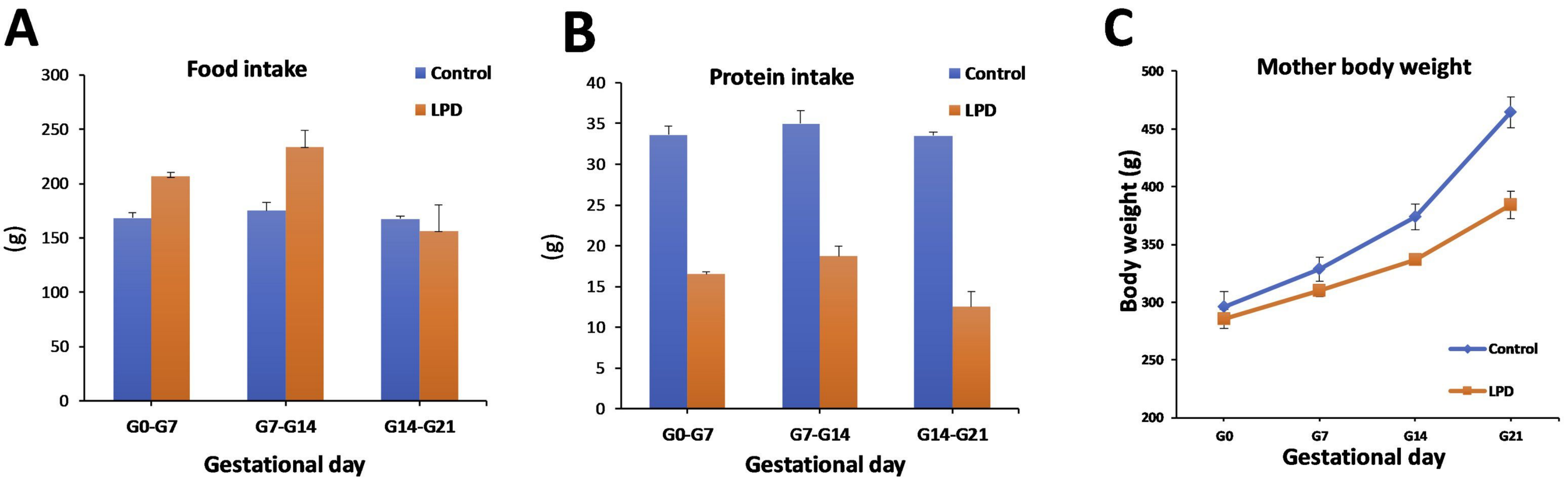

Supplement: Supplementary file 1 [file nutrients-13-03719-s001.zip › Supplementary materials.pdf]
